# Improving the Reporting of Young Children’s Food Intake: Insights from a Cognitive Interviewing Study with Mothers of 3–7-Year Old Children

**DOI:** 10.3390/nu12061645

**Published:** 2020-06-02

**Authors:** Dorota Zarnowiecki, Rebecca A. Byrne, Glen E. Bodner, Lucinda K. Bell, Rebecca K. Golley

**Affiliations:** 1 School of Pharmacy and Medical Sciences, University of South Australia, Adelaide, SA 5000, Australia; lucy.bell@flinders.edu.au (L.K.B.); rebecca.golley@flinders.edu.au (R.K.G.); 2 NHMRC Centre of Research Excellence in the Early Prevention of Obesity in Childhood, Sydney School of Public Health, University of Sydney, Sydney, NSW 2006, Australia; 3 Caring Futures Institute, College of Nursing and Health Sciences, Flinders University, Bedford Park, SA 5042, Australia; 4 Institute of Health and Biomedical Innovation, Centre for Children’s Health Research, Queensland University of Technology, Brisbane, QLD 4101, Australia; ra.byrne@qut.edu.au; 5 College of Education, Psychology and Social Work, Flinders University, Bedford Park, SA 5042, Australia; glen.bodner@flinders.edu.au

**Keywords:** child food intake, dietary intake assessment, short food questionnaire, cognitive interview, qualitative research, parent

## Abstract

Short food questions (SFQ) allow for rapid reporting of food intake across a variety of settings but are limited by poor validity and reliability. Understanding the recall process used by parents to report children’s food intake can improve question design and psychometric performance. This study aimed to improve understanding of how parents report children’s dietary intake using SFQ. Semi-structured, cognitive interviews were conducted with 21 mothers of 3–7-year-old children. Mothers were asked to ‘think-aloud’ while answering SFQ about their child’s food intake. Thematic analysis identified themes relating to parent’s question and answer process and barriers to recall. Information retrieval strategies focused on ‘use-of-time’ and ‘sphere of food provision’ and differed for core versus unhealthy foods. Recall of routine and home food provision were used to report core food intake, whereas recall of special occasions and food provision outside the home guided recall of discretionary foods. Mothers utilize different recall strategies for core and discretionary foods based on use of time and the sphere of food provision. The ease of reporting children’s dietary intake may be improved by utilizing a shorter recall time frame, clear and direct question wording, and use of food examples and recall prompts.

## 1. Introduction

Childhood overweight and obesity is a global public health issue [1]. Dietary intake plays an integral role in the prevention of obesity in children, and modification of dietary intake is a key intervention strategy [2]. Accurate measurement of dietary intake is essential for evaluating nutrition and obesity intervention programs, monitoring trends in dietary behaviours, and informing policy and practice decisions. There are many challenges to measuring dietary intake in young children, particularly in the policy and practice context. Research and evaluation in this area is limited by a lack of validated and ‘fit for purpose’ brief tools for measuring young children’s food intake [3]. Traditional dietary assessment methods such as diet records, 24-h dietary recalls and food frequency questionnaires have been extensively evaluated and can capture total dietary intake with reasonable accuracy [4,5]. However, these methods are long, burdensome for participants and costly, limiting their usefulness within policy and practice settings [4]. In comparison, short food questions (SFQ) are an appealing and viable approach to monitor population dietary intake and evaluate public health nutrition programs [6]. SFQ can be tailored to dietary outcomes of interest, support rapid reporting of food intake, are quick to administer in a variety of formats and have low respondent burden.

Reviews of SFQ measuring food intake in children and adolescents show that no single set of short questions provides both reliable and accurate estimates of intake across a range of food groups [3,6]. Differences in intake reported in SFQ compared with 24 h recalls range from under-reporting of vegetables to over-reporting of grains, meat and dairy [7]. Reasons for the differential performance of food groups are not well understood, but may arise due to the variability and frequency of food intake, or due to social desirability [8]. The accuracy of dietary intake reported in SFQ is also reliant on respondents’ memory and ability to report frequency and estimate quantities [9]. Drawing on theories that underpin questionnaire completion, memory, and frequency estimation strategies may provide direction for making SFQ easier for respondents to complete, ideally while also increasing their accuracy.

Response errors arise when respondents misinterpret questions, have difficulty retrieving information from memory, misjudge responses, or edit responses due to influences such as social desirability [10,11,12]. Response biases can be affected by the questionnaire design, context within which questions are administered and respondent’s own characteristics [10]. When reporting frequency, respondents typically generate estimates using cognitive heuristics (i.e., mental shortcuts) rather than enumerating specific instances (i.e., counting) [8,13,14], sometimes based on general impressions rather than memory recall [8]. The choice of response strategy can also introduce systematic estimation biases [14]. These biases are unlikely to be identified in traditional approaches to questionnaire measurement, which focus on detecting overt problems with questionnaire administration, such as length, readability, comprehension and ease of administration [12]. 

Cognitive interviewing allows direct study of the question and answer process used by respondents when completing SFQ, providing an understanding of a questionnaire from the respondent’s perspective [12,15]. Cognitive interviewing has been used primarily to refine question wording and readability, and improve face validity of dietary intake questionnaires [16,17,18,19]. Cognitive interviewing approaches can also be used to understand information processing, recall strategies and response estimation strategies [17,20,21]. The present study used a cognitive interviewing approach to: (1) understand how SFQ are interpreted, (2) identify the recall process and response strategies parents use, and (3) identify barriers and facilitators experienced by parents when reporting their children’s dietary intake. The overall aim was to improve understanding of how parents report dietary intake of their 3–7-year-old children. The age group of 3–7 years was chosen to align with two key periods of childhood: the first 2000 days (up to 5 years) when many dietary habits are established; and the first years of primary school (i.e., junior primary school) when declines in children’s diet quality occur [22]. Therefore, children of this age group are a key target for nutrition interventions, necessitating well-designed SFQ for use in evaluation of public health nutrition programs. 

## 2. Materials and Methods 

This study used semi-structured cognitive interviews, combining think-aloud and retrospective probing question approaches to evaluate question comprehension, response and estimation strategies, and other factors influencing responses to SFQ completed by parents of 3–7-year-old children. Thinking aloud involves participants vocalizing their thoughts out loud while concurrently undertaking a task, such as completing a questionnaire [20,21]. The study was conducted in accordance with the Declaration of Helsinki, and The University of South Australia Human Research Ethics Committee approved the study protocol (200014). All participants provided written informed consent and verbal assent prior to commencing recording of interviews. The study was registered with the Australian New Zealand Clinical Trials Registry (ACTRN12617001091392).

### 2.1. Participants and Recruitment 

Parents of children aged 3–7 years were recruited from August to November 2017 via flyers at university campuses, childcare centres, kindergartens and social media, utilising snowball recruitment strategies. Participants self-selected to be involved by contacting the researchers via email or telephone. Primary caregivers responsible for food provision with sufficient English fluency to undertake the interview were eligible. Parents of children with health conditions considerably affecting their food intake (i.e., Type 1 diabetes, coeliac disease) were excluded. One parent of a child with a milk allergy was included as dairy was not one of the food groups evaluated, whilst none were excluded due to health conditions. The interview protocol was pilot tested with two parents and no alterations made; therefore, pilot interviews were included in the main analysis. Cognitive interviewing guidelines recommend a sample size of 5–15 participants per interviewing round [21]. Each of the four food groups evaluated were considered an interviewing round as it was hypothesized that parents may use a different recall approach for each food group, giving a required sample size of 20–60 participants. Interviews were conducted until data saturation was reached within this range, indicated by the emergence of no further new themes [23] after review of summary notes taken in the first 16 interviews. Participants who completed the interview received a pamphlet providing nutrition advice based on the Australian Dietary Guidelines [24] and were entered into a random prize draw to win one of 10 cookbooks (valued at approximately USD$25). This strategy was employed to acknowledge time taken to be involved in the study and as a strategy to recruit a more diverse sample and reduce selection bias.

### 2.2. Short Food Questions (SFQ)

SFQ measuring intake were evaluated measuring intake of: (1) discretionary foods (defined according to Australian Dietary Guidelines as ’foods which do not fit into the Five Food Groups, not necessary or essential for a healthy diet, and are high in saturated fat, added sugars, salt and/or alcohol and low in essential nutrients and fibre’ [25]), and three of the Five Food Groups (referred to as ‘core food groups’); (2) grains and cereals; (3) meat and alternatives; and (4) vegetables. These food groups have poorer statistics in validation and reliability studies than other food groups, suggesting that they are harder for parents to answer [6]. Additionally, a recent systematic review identified that questions about meat and alternatives and grain-based foods were less commonly evaluated, and their performance tended to be inferior [6]. Questions from three established, age-appropriate and psychometrically evaluated questionnaires were used (Table 1): (1) ‘Child Diet Questionnaire’ (CDQ) measuring obesity-related food intake over the previous seven days in 4–16 year old children [26]; (2) ‘Pre-schooler Dietary Questionnaire’ (PDQ) measuring dietary ‘risk’ in 3–5-year-old children over the previous seven days [27]; and (3) ‘Short Food Survey’ (SFS) measuring usual food intake and diet quality over the previous few months in 4–11-year-old children [7]. These questionnaires were selected as all were developed and validated for use with parents of Australian children, and therefore used consistent definitions for the four food groups according to the Australian Dietary Guidelines [24]. Further, the question design differed with regards to measurement of portion size, response options, wording and structure (i.e., detail/prompts within question) and time frame (usual intake versus previous seven days) (Table 1). For the PDQ, the only tool assessing portion size, parents reported the amount their child consumed per intake frequency, using provided response options representing small, medium and large portion sizes, using cooking measurements. This provided insights into the recall process and parent-reported barriers to reporting children’s questions across a range of SFQ approaches. 

To reduce interview length and participant burden, all participants answered questions about discretionary food intake and were randomly allocated to answer questions from two of the three core food groups. Randomization of food groups and questionnaires to ID numbers was conducted prior to commencing the study pilot using random number generation in Microsoft Excel^TM^. ID numbers were firstly randomly allocated to core food groups, and then food groups were randomly allocated to a questionnaire. Lastly, the order of food groups presented was randomized. Randomization was counterbalanced to ensure a relatively even spread of food groups and questionnaires (Table 1). Participants were allocated to an ID number in sequential order of recruitment into the study following eligibility screening. Two vegetarian parents/children were assigned to the next sequential condition that did not include the meat and alternatives group.

### 2.3. Study Protocol

The study was conducted at the University of South Australia, in Adelaide, South Australia. Interviews (30 to 72 min, average 51 min) were conducted in-person (*n* = 15) or via Skype (*n* = 6) for those residing outside of Adelaide. Interviews via Skype overcame constraints associated with location, time and carer duties that may have prevented parents from undertaking interviews. Skype rather than telephone interviews were used to mimic an interview setting as closely as possible. The interaction between interviewer and interviewee and presence of nonverbal cues in a Skype interview are comparable to face-to-face interviews [28]. Skype participants were emailed instructions and questionnaires prior to their interview and were instructed not to read or complete the questions prior to the interview. In all other aspects, the protocol for Skype interviews was identical to face-to-face interviews. Prior to commencing the interview, the procedure was explained, and participants confirmed their consent for the interview to be audio-recorded. The think-aloud approach was explained and practice of think-aloud was undertaken using a non-food related topic.

Cognitive interviews were conducted by one interviewer (D.Z.) with experience in semi-structured interviewing. A protocol for the cognitive interviews and standardised interview guide were developed using the methodology of Willis [21]. A combination of think-aloud and retrospective probing approaches were used [21]. First, parents were instructed to think-aloud while completing SFQ about their child’s food intake, vocalizing their thoughts as they responded to the question. Less vocal participants were prompted during the think aloud using scripted prompts (i.e., ‘What are you thinking?’). This approach enabled parent-defined issues about question comprehension, recall and estimation processes to be identified. Next, parents were asked retrospective probing questions, using a semi-structured guide (Appendix A) in combination with spontaneous prompts as relevant themes emerged, to reveal further insights into their response process. Probing questions asked about comprehension, recall strategies (i.e., “How did you determine [X]?”), memory prompts (i.e., “What did you think about when trying to recall…?”), ease/difficulty of coming to a response, confidence in answer, comprehension, and suitability of response options. Where a participant verbalized more details in the think-aloud, fewer probing questions were asked. This process was completed for the first food group, and then repeated for the other assigned foods groups. Lastly, participants completed a questionnaire about basic demographic characteristics of the child and family.

### 2.4. Data Analysis

The audio-recorded interviews were transcribed verbatim by a professional transcription company and/or research staff in de-identifiable format, and imported into NVIVO software (Version 11) [29] for analysis. All transcripts were checked for accuracy by the interviewer after transcription. Analysis and coding were conducted to: (1) understand the recall processes used by parents and parent-reported barriers and facilitators to recall; and (2) compare response estimation strategies used by parents when reporting intake of the evaluated food groups. An a priori list of coding themes was drafted by two researchers (D.Z. and L.K.B.), one of whom was the interviewer, based on the interview guide and written notes taken during 16 interviews. Pilot coding of the first two completed interviews was conducted by three researchers (D.Z., R.A.B., L.K.B.) [30]. Pilot coding by the three researchers was compared and discussed to develop a coding approach and thematic outline against which the remaining interviews were coded by one researcher (D.Z). Thematic analysis combining a deductive and inductive approach was used to code interviews and identify patterns arising within the data [30,31]. Four additional themes for parent-reported barriers and facilitators of reporting child intake emerging during coding [30]. Themes relating to parents’ recall process were interpreted within the ‘Question and Answer’ model [12], a model of the survey response process derived from cognitive psychology. Use of a framework to classify themes addressed concerns about objectivity raised within analysis of cognitive interview data [15]. As the three core food groups were not all evaluated in every interview and fewer items were assessed, they were combined as ‘core foods’ for analysis to enable comparison with themes identified for discretionary foods. This equalized the number of interviews and items for core foods and discretionary foods. Throughout coding and analysis, reflexive discussion of coding and themes was conducted by the research team to reduce subjectivity and bias in analysis and interpretation of themes [32]. 

Response strategies used by parents to report their child’s food intake were coded using the taxonomy developed by Conrad et al. [8] based on earlier work [13,14]. Two coders were trained (by G.E.B.) to identify examples of each of five response strategies vocalized by parents when estimating their child’s food intake. For each food group in each interview, a coder reviewed the think-aloud section of the transcript for instances of each response strategy and pasted the best example of each strategy from the transcript into a spreadsheet. Between zero and five response strategies were coded for each interview both per food group and per questionnaire. Thus, for each food group/measure a parent may have used up to five response strategies. Given the small sample, analysis was conducted by comparing frequency of responses coded for each response strategy. Using a sample interview, the raters agreed on the presence or absence of each response strategy in the sample transcript in 12 of 15 cases (agreement = 80%) and disagreements were resolved via discussion. The third author (G.E.B.) reviewed the final spreadsheet to ensure that the transcript excerpts reflected the indicated strategy.

## 3. Results

### 3.1. Participant Characteristics

Twenty-one interviews were completed. All participants were females (hereafter, mothers), aged 29 to 44 years (mean age 36.2 ± 3.7 years) with children (60% female) of mean age 4.3 ± 1.2 years. Most participants (81%) were from South Australia. The majority (81%) had completed tertiary or postgraduate university education (Table 2). Skype participants (*n* = 6; 29% of sample) were of similar mean age to face-to-face participants, socioeconomic status (based on postcode), marital status and ethnicity. Compared with face-to-face participants, a smaller proportion of Skype participants had completed a postgraduate university degree (33% vs 54%) and none were employed full-time (versus 27% of face-to-face participants).

### 3.2. Recall Process Used to Report Children’s Intake

**Figure 1:**
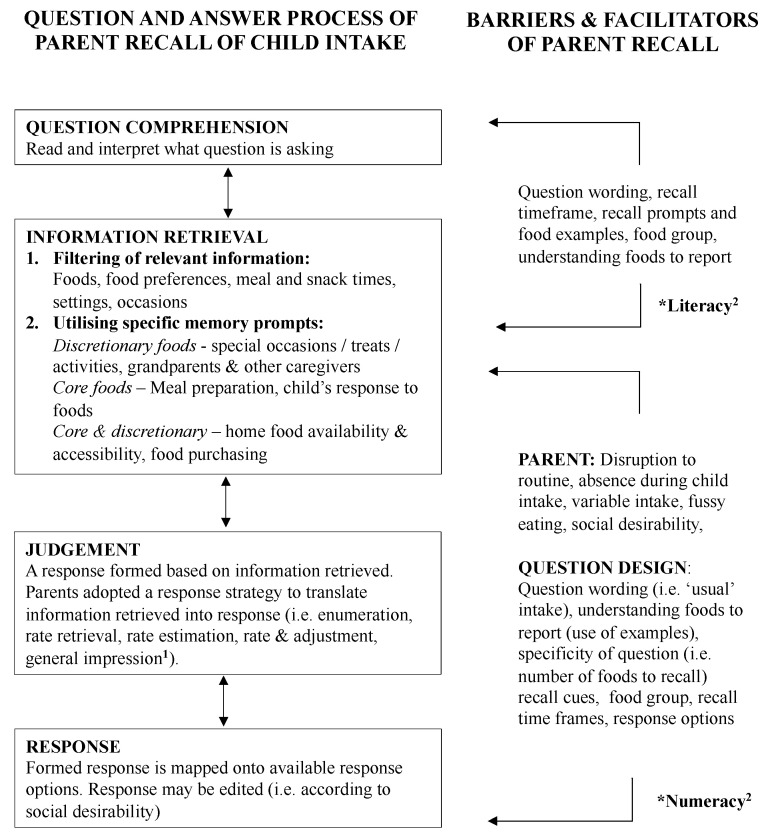
Recall process used by mothers (*n* = 21) to report children’s dietary intake, informed by the Question and Answer model described by Collins et al. [12]. (1) Response strategies were classified according to the taxonomy described by Conrad et al. [8]. (2) Literacy and numeracy are hypothesized to impact on question comprehension and response, but were not captured in themes arising from interviews, likely due to the high proportion of participants with tertiary education level or higher.

The recall process used by mothers to report children’s food intake aligned with the response process described by Collins’ ‘Question and Answer’ model [12] (Figure 1). Mothers first attempted to comprehend what the question was asking, based on their interpretation of the terminology used and foods listed in the question. They then attempted to retrieve relevant information from memory by adopting a recall strategy and utilising specific memory prompts. Next, mothers used a variety of response strategies to filter their recalled information into a response and lastly formatted their response to fit with the response options. Notably, this process was not linear: mothers moved back and forth between these steps to generate their final answers. 

### 3.3. Information Retrieval

#### 3.3.1. Step One—Filtering Relevant Information

Information retrieval appeared to be approached in two steps. First, mothers used filtering processes to identify which foods, mealtimes and settings were most relevant to their child/family and ruled out irrelevant information (Table 3). Consideration was given to child and family food preferences, usual routine and occasions or settings where foods were consumed. Food preferences were used to identify which foods needed further consideration versus which foods were disliked or not consumed and could be eliminated from further consideration. Routine was used as a recall prompt in a different manner for core (grains, meat and vegetables) versus discretionary foods. When recalling core food intake, the ‘usual’ family routine was considered in terms of the typical weekly schedule of activities, care arrangements of the child (i.e., grandparents, childcare, school) and meals provided. Conversely, when recalling discretionary foods mothers considered whether the usual routine had been disrupted, including due to special events or activities. Differences were also noted in how meal and snack times were used as retrieval cues for core and discretionary foods. For core foods, recall focused on intake at main meals (breakfast, lunch, dinner) whereas for discretionary foods, mothers more so considered snack times and intake outside of main meals.

#### 3.3.2. Step Two—Use of Specific Memory Prompts

In the second step of information retrieval, specific memory prompts were utilized to retrieve information about foods consumed. Memory prompts differed for core versus discretionary foods and centred around ‘use of time’ or the ‘sphere of food provision’ (Figure 1). ‘Use of time’ recall strategies considered the family’s everyday routine versus occasions that occurred outside of usual routine such as social events or family activities. ‘Sphere of food provision’ strategies considered whether provision occurred by parents within the home (related to everyday routines) or outside the home, and by other caregivers. For core foods, recall was cued using everyday routine and food provision within the home, whereas for discretionary foods, recall was cued using occasions/activities occurring outside of routines and provision outside the home and by other caregivers. 

Occasions, events, treats and activities: One of the most prominent recall strategies employed to recall intake of discretionary foods was consideration of special occasions and activities. These included school holidays, family time, weekend or after-school treats, celebrations, and family or friend get-togethers.

Grandparents and other caregivers: A prevalent setting for consumption of discretionary foods was noted to be when children were cared for by their grandparents. Mothers used time spent with grandparents as an anchor to recall the type, frequency and quantity of discretionary foods consumed. Several mothers also noted that the other parent (father) provided discretionary foods when picking up the child from childcare, school or extra-curricular activities, and used this as a prompt for recall. A few mothers also recalled food provision by grandparents or other caregivers in relation to core foods. 

Meal preparation: Mothers recalled meal preparation and foods their children were offered (i.e., sandwich, popcorn, cut up vegetables), largely when recalling intake of core foods. This was typically combined with consideration of their child’s reaction to the foods offered. 

Children’s response to food: Mothers recalled their child’s reaction to foods to bridge the gap between provision and intake, especially for core foods. Mothers considered how often they served the food and then adjusted their response by considering whether their child ate it, left it on their plate, picked it out of the meal or threw a tantrum. Some noted that their child went through phases of food intake and used the current phase as a recall cue.

Home food availability and accessibility: Food availability at home cued recall of core foods within the context of everyday routine, by considering foods available in the fridge/pantry. Availability of discretionary foods at home and consideration of which discretionary foods the child was able to help themselves to (accessibility) were both used to recall discretionary food intake. 

Food purchasing: To cue recall of core foods consumed, some mothers recalled foods purchased during grocery shopping. When recalling discretionary food intake, they also considered other locations of purchase (i.e., sporting club).

### 3.4. Response Strategies Used to Estimate Food Intake

**Figure 2:**
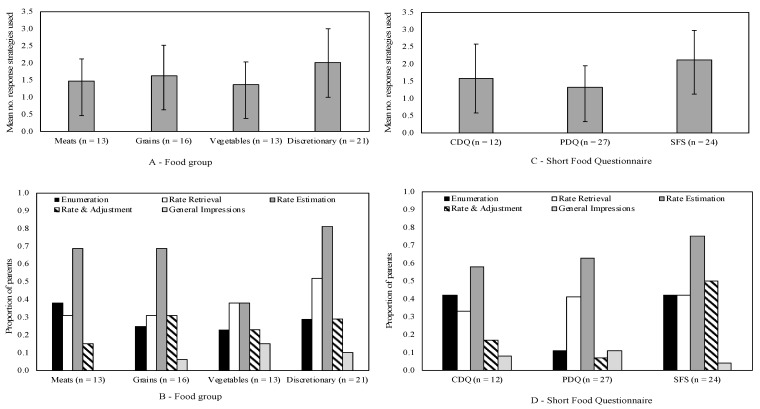
Mean number of response strategies and proportion of Australian mothers of 3–7 year old children (*n* = 21) using each response strategy when reporting their children’s dietary intake using short food questions: (**A**) Mean number of response strategies used by mothers by food group; (**B**) Proportion of mothers using each response strategy by food group; (**C**) Mean number of response strategies used by mothers by short food questionnaire; (**D**) Proportion of mothers using each response strategy by questionnaire. Note: Numbers presented in parentheses in x-axis represent the number of parents who completed questions for the food group (**A**,**B**) and the questionnaires (**C**,**D**).

On average mothers used more than one response strategy to estimate their child’s food intake for each food group and measure (Figure 2, Table 4). In general, the distribution of response strategies was similar across food groups (Figure 2B). All five response strategies were used to some extent across the four food groups, with the most common response strategy used being rate estimation (64%). The tendency for mothers to use more than one response strategy was also consistent across measures and was most pronounced for the SFS (Figure 2D). Across measures, rate estimation was the most likely response strategy. A rate and adjustment strategy was used (numerically) more often with the SFS measure than the other two measures. In contrast, an enumeration strategy was used (numerically) less often with the PDQ measure than the other two measures.

### 3.5. Barriers to and Facilitators of Reporting Children’s Dietary Intake

Identified barriers to recall were predominantly related to questionnaire design, with a small number of parent-related factors impacting question response. Elements of question design, such as the wording, examples used, recall timeframe and response options, impacted how mothers approached the question and answer process. Conversely, question specificity and use of examples were noted as facilitators that improved ease of reporting. 

Understanding of foods to report: Misunderstanding which foods to include within the question was common. Mothers were heavily guided in which foods they should report by the examples provided in the question. Examples also assisted mothers when they did not understand the terminology used. A point of confusion for several mothers was whether to include homemade versions of discretionary foods (e.g., ice blocks, hot chips), which they felt were healthier than store-bought versions. 

Specificity of questions: Most mothers preferred questions to provide detailed examples of which foods to include (and exclude). Examples served as a recall prompt and mothers tended to only recall foods provided in examples, demonstrating the importance of using appropriate examples to capture all relevant foods. A few mothers also noted preferring defined questions that asked about a smaller number of foods.

Reporting portion size: The primary approach used to estimate portion size was to consider how the food was served and translate this to fit the response options. Some mothers noted visualizing how the food would fit within the provided measures. Mothers found it more challenging to estimate portion sizes in grams and centimetres, and preferred estimates based on cooking measurements, such as cups, tablespoons or number of items, or how the food is typically served (e.g., a slice of ham, a cup of popcorn). When asked to provide a response in units (e.g., grams/centimetres), mothers attempted to convert their response from how the food was served, which required numeracy skills and a greater cognitive load, particularly when the unit recalled did not fit with provided response options. Some noted they did not keep track of the amount of food their child consumed, particularly vegetables and discretionary food, and therefore ‘guesstimated’ portion size. 

Recall time frame: Mothers preferred to recall their child’s intake over a shorter recall timeframe of up to seven days. When asked to report intake over a longer time period, mothers still tended to recall the past week. However, intake over a longer period (i.e., past month) was considered for foods consumed less frequently, especially discretionary foods.

Recall difficulties: Many mothers had difficulty recalling elements of their child’s intake across all food groups. Some focused on recalling intake at the primary meal when the food was consumed (i.e., meat at dinner) without thinking about other times when the food might have been consumed. To account for recall difficulty, mothers reported ‘usual’ intake or erred on the side of caution by increasing their estimate to compensate for potential omissions. 

Unsure of foods child consumes when away from parents: The most notable parent-related recall barrier was a lack of knowledge of what the child consumed when not in the care of the parent (e.g., at childcare, with another caregiver, at social occasions). Mothers either excluded their child’s intake at these occasions from their estimate, or guesstimated their intake. 

Frequency versus portion size: Some mothers were concerned that reporting only the frequency of intake for certain foods (e.g., vegetables, lollies, chocolate) may not accurately reflect their child’s consumption. When their child consumed a small amount of a food (less than what would be considered a serve) but over several days, mothers were concerned that their child’s reported intake appeared inflated. 

Child fussy eating: Most mothers referred to their child’s fussy eating and the challenge it presented for recalling intake. They found it easier to report what was offered compared to what and how much their child ate. 

Reporting ‘usual’ intake: Terminology relating to ‘usual’ intake in the questions presented a challenge, as mothers experienced a tension between wanting to report what they perceived was their child’s ‘best’ usual intake versus ‘actual’ intake. This was also reflected in social desirability, described below. 

Social desirability: Social desirability was perceived to be demonstrated when mothers used language that placed value judgements on their child’s intake. For instance, too much discretionary food or too little core food was described as ‘bad’, and in some instances perceived acceptable intake was described as ‘good’. Mothers demonstrated a discomfort and a desire to focus on their child’s usual intake of ‘good’ foods versus their atypical intake of ‘bad’ foods. Some noted that it was confronting to answer questions about their child’s intake as it highlighted a disparity between what their child ate versus what they wanted their child to be eating, which in turn elicited feelings of guilt. 

## 4. Discussion

This study used a cognitive interviewing approach with mothers of 3–7-year-old children to examine how parents report their children’s dietary intake, with an aim to identify opportunities to improve short food questions (SFQ). The identified response process reflected steps of the ‘Question and Answer’ process described by Collins [12]. Mothers attempted to understand what the question was asking, retrieved relevant information from long-term memory, processed information, decided how to form a response and employed a response strategy for reporting their answer. Mothers processed questions about core and discretionary foods differently, structuring their recall according to ‘use of time’ (i.e., food consumption as part of the family routine or at special/social occasions) and the ‘sphere of food provision’ (by parents at home versus childcare, or by other carers outside of the home). Mothers typically used more than one response strategy to come to their answer, demonstrating the complexity of reporting a frequent behaviour such as dietary intake. 

Cognitive interviewing has been used previously to improve readability and wording of questionnaires measuring adult food intake [19,33,34], parent report of children’s intake [35,36], feeding practices [37] and feeding difficulties [38]. This study adds to this body of literature by using cognitive interviewing to understand recall processes used by parents when reporting children’s intake. Johnson-Kozlow [17] identified eight categories of recall strategies used by adults completing a food frequency questionnaire, including rules and routines, eating schedule, evaluation of foods as ‘good’ or ‘ bad’, food preparation, setting where food was purchased, events and people. Consistent with our findings, a number of these strategies utilized routine and context of the eating occasion, including the setting and the people present [17]. However, the recall process used to recall adults’ own intake had a strong evaluative component that classified foods as good or bad and considered the individual’s own food consumption rules (i.e., ‘I never eat’) as a recall strategy [17]. Although some evaluative comments were made by mothers in this study, recall of children’s dietary intake was driven more so by episodic memory, including recall of the context surrounding food intake, mothers’ experiences of the mealtime (i.e., child fussiness and response to food), and the setting and people present. Thus, mothers appear to use a different approach to recall their children’s intake compared to their own intake.

To improve understanding of why differential misestimation occurs for food groups (i.e., overestimation of grains, meat and vegetables and underestimation of discretionary foods) the present study evaluated response strategies used by parents to estimate food intake [8]. Response strategy choice depends on the likely frequency of the behaviour, and each strategy has a signature weakness [8,13,14]. Enumeration, more commonly used when behaviour frequencies are lower, typically leads to underestimation because some instances are likely to be missed or forgotten [14]. Conversely, estimation strategies are commonly used when frequencies are higher, and typically lead to overestimation when exceptions are not taken into account [14,39]. Dietary intake is a frequently occurring behaviour, thus mothers were particularly likely to use rate estimation, a pattern observed across core (but interestingly less so for vegetables) and discretionary food groups, and across all evaluated questionnaires. This reliance on estimation strategies relative to enumeration across food groups and measures suggests that overestimation of food intake may be a more common issue than underestimation [14]. However, replication with larger samples is needed to confirm whether response strategies used vary across food categories and/or measures. Question wording may also lead participants towards use of particular response strategies [39]. For instance, a rate and adjustment strategy was used (numerically) more often with the SFS, perhaps due to question wording (‘usual intake’) and longer recall time period (past month). In contrast, an enumeration strategy was used (numerically) less often with the PDQ measure than the other two measures.

Errors may be introduced at each stage of the question and answer process. Respondents may interpret the question differently to how it was intended or misunderstand terminology about what to include in their response [12]. For example, in this study there was confusion about reporting homemade versions of discretionary foods. Information retrieval may be affected by whether the information was stored in long-term memory, if the context of recall differs to that of storage, and how easy it is to distinguish the memory from similar events [12]. When lapses in memory occur, participants compensate for incomplete information by employing estimation strategies. Frequency estimates are often based on the ease of retrieving one or more specific instances from memory [40]. Hence, use of estimation strategies introduces cognitive heuristics and biases that can result in systematic errors. The regularity of a behaviour or event impacts on the ease with which it can be recalled and the response strategy employed [8,39]. Less frequent but regular behaviours are more distinct and easier to recall and enumerate compared with frequent or highly variable behaviours [39]. This may affect reporting of core food groups which are consumed with high frequency across meals and snack occasions, with likely variability in types of foods consumed across eating occasions. To overcome this, mothers utilised recall prompts related to ‘routine’ and the ‘sphere of food provision’ to report intake of frequently consumed core foods. A frequently used recall prompt for discretionary foods was time spent with other caregivers, which is a common source of error in proxy reporting as this intake is not witnessed. This study provides insights into how this proxy recall is achieved. A potential strategy to overcome this is the use of checklists for other caregivers. Finally, to provide their answer, respondents adapted their responses to fit the provided response options and further modified their response to meet perceived researcher expectations or social desirability [12]. 

### Strengths and Limitations

This is one of few studies [17,41] to use cognitive interviewing to evaluate recall processes used when reporting dietary intake. This study builds on these previous studies that examined the recall process in children and adults reporting their own intake, by examining how parents’ proxy recall children’s dietary intake. A strength of this study was the evaluation of SFQ measuring core and discretionary food groups, which provided insights into differing recall approaches used for core versus discretionary foods. Three different questions of styles, with differing timeframes and response options, were evaluated, informing recommendations for future question wording and design. To reduce the possible effect of individual variation in ability to verbalize thoughts, participants practiced the think-aloud prior to commencing the interview and the order of food groups and questionnaires were randomized to reduce learning effects. However, the cognitive interviewing process itself may affect how respondents answer questions. Our sample size was small, limiting statistical comparisons of response strategies which could be undertaken. Larger studies would enable further analysis of response strategies for food groups and question wording, to explore how question wording influences response strategies used. Despite recruitment attempts via diverse modes, participants were mostly of higher socioeconomic status with over two-thirds completing a University degree, limiting the generalizability of findings. In particular, it was not possible to evaluate the impact of low literacy and numeracy on the results. Verbalizing thoughts such as is required in cognitive interviewing does not come naturally and less articulate respondents may be disinclined to participate. Finally, all participants were female, which reflects women’s ongoing role as primary food providers in families [42]. Further research should explore the recall processes used by fathers and other caregivers, as well as in vulnerable and ethnically diverse populations. 

An inherent challenge in reporting dietary intake is that eating is a frequent behaviour and therefore presents a task with a high cognitive load for respondents to recall, which further increases when parents report intake for their child. Questionnaire design and accuracy can be improved by reducing the cognitive load, and as such SFQ provide a promising approach to measuring children’s dietary intake with greater ease. Findings of this study suggest that parent reporting of children’s food intake using SFQ may be optimized by use of a shorter recall timeframe (up to seven days), brief and direct question wording and use of examples and prompts to aid recall. Digital delivery of SFQ (i.e., via websites or Apps) provides an opportunity for incorporating more sophisticated prompts, contextual cues, instructions and food examples, without increasing question length, and may enable collection of information from multiple sources involved in the care of the child. Our findings suggest that parents find question terminology referring to reporting ‘usual’ intake troublesome. Subtle changes to language, such as omitting the phrase ‘in the past’ and use of short declarative statements over longer interrogative versions (i.e., “How often does your child usually eat…”) may be optimum [35,36]. Dickin [35] found that parents wanted simple wording and brief items, but also wanted examples of foods to clarify meanings, without which some parents underestimated intake. Further, the inclusion of use of time, setting and contextual recall prompts which are specific to core versus discretionary foods may also aid parents’ recall [43]. Use of visual aids can enable the inclusion of prompts and examples without increasing question length, to ensure the suitability of SFQ for use with individuals of lower literacy and numeracy [36]. 

## 5. Conclusions

This study used a cognitive interviewing to provide insights about the recall process used by mothers when reporting their children’s food intake using SFQ. Drawing on cognitive theories underpinning questionnaire completion, memory and frequency estimation strategies, this study informs how the design of SFQ can be optimized to improve parent reporting and ease of completion. Mothers utilized different information retrieval strategies for core and discretionary foods based on use of time and the sphere of food provision. When reporting intake, more than one response strategy was used, most frequently estimation, which may be associated with overestimation. These findings reflect the high cognitive load of reporting a highly frequent behaviour such as dietary intake. The ease of reporting children’s dietary intake by parents may be improved by utilising a shorter recall time frame, clear and direct question wording, and use of food examples and recall prompts. Further studies to understand recall processes used by parents of lower education, income and of differing ethnicity are needed to inform how design of SFQ can be improved for these populations.

## Figures and Tables

**Table 1:** Questionnaires and questions evaluated in cognitive interviews with Australian mothers (*n* = 21) to understand recall process used when reporting 3–7-year-old children’s dietary intake.

	Child Diet Questionnaire (CDQ) [26]	Pre-Schooler Dietary Questionnaire (PDQ) [27]	Short Food Survey (SFS) [7]	TOTAL (n)
Meat and alternatives	N/A	2-items: How many times in past 7 days and how much (per time) did your child consume: Red meat; Fresh fish. *n* = 7	4-items: How often does your child usually have: Red meats; White meats; legumes or meat alternatives; Eggs. *n* = 6	13
Grains	N/A	1-item: How many times in past 7 days and how much (per time) did your child consume: Grains (not including bread, noodles or pasta) *n* = 8	3-items: How often does your child usually have: Bread; Pasta, rice, noodles or other cooked cereals; Breakfast cereals *n* = 7	15
Vegetables	3-items: Vegetables (cooked or raw) your child has eaten over past 7 days; How often has your child had vegetables past 24 h; How many different vegetables past 24 h *n* = 5	3-items: How many times in past 7 days and how much (per time) did your child consume: Green and brassica vegetables; Orange vegetables; Other vegetables *n* = 5	3-items: How often does your child usually have: Starchy vegetables; Salad vegetables; Cooked vegetables *n* = 4	14
Discretionary foods	13-items: How many times has your child eaten these foods/drinks over past 7 days: Peanut butter/Nutella; Pre-sugared cereal; Sweet biscuits, cakes, etc; potato crisps, savory biscuits; lollies, muesli bars; Chocolate; Soft drink/cordial; Ice-cream; Pie, pasty, sausage roll; Pizza; Hot chips; Processed meats; Takeaway *n* = 7	6-items: How many times in past 7 days and how much (per time) did your child consume: Chips, pop-corn; hot potato products; meat products; sweet biscuits, cakes etc; chocolate; ice-cream *n* = 7	10-items: How often does your child usually have: Soft-drink, cordial, sports drinks; Fruit drinks; Takeaway; Hot potato products; savoury snacks; Sweet biscuits, cakes. etc; savoury pastries; Chocolate/lollies; Ice-cream; Meat products. *n* = 7	21
Response options:	Tick Yes/no; OR Number of times Nil-5+	Nil, 1, 2–4, ≥5 times AND Nil—specified portion size consumed per time (cooking measurements e.g., cup, tablespoon, number of slices/pieces)	How often: doesn’t eat, each day, each, week, each month, AND how many times per frequency	
**TOTAL (n)**	**12**	**27**	**24**	

‘n’ represents the number of participants who completed questions from the questionnaire and food group.

**Table 2:** Descriptive characteristics of mothers of 3–7-year-old children participating in the cognitive interviewing study (*n* = 21).

	Parent	Child
Age (years) *	36.2 ± 3.7	4.3 ± 1.2
Gender—Female (%)	100.0 (21)	57.1 (12)
Number of children in family (%) *		
One child	35.0 (7)	
More than one child	65.0 (13)	
Marital status—married/living with partner (%) *	90.5 (19)	
Ethnicity—Caucasian (%) *	100.0 (20)	
Australian State of residence (%)		
South Australia—Adelaide	71.4 (15)	
South Australia—Rural	9.5 (2)	
Australian Capital Territory	9.5 (2)	
Queensland	4.8 (1)	
Tasmania	4.8 (1)	
Socioeconomic status (SEIFA decile)		
Low (decile 1–3)	28.6 (6)	
Middle (decile 4–7)	23.4 (5)	
High (decile 8–10)	47.6 (10)	
Education level (%) *		
Completed high school	4.8 (1)	
Trade certificate or diploma	9.5 (2)	
University or tertiary qualification	33.3 (7)	
Postgraduate university degree	47.6 (10)	
Employment status (%) *		
Full-time employment	19.0 (4)	
Part-time employment	47.6 (10)	
Self-employed	14.3 (3)	
Home duties	9.5 (2)	
Student	4.8 (1)	
Child dietary restrictions (%)		
None		81.0 (17)
Vegetarian child		4.8 (1)
Vegetarian mother (no meat at home)		4.8 (1)
Peanut allergy		4.8 (1)
Avoid dairy		4.8 (1)

Data presented as mean ± standard deviation for age, and percentage (number of participants) where denoted (%); * Missing data for *n* = 1 who did not return demographic questionnaire.

**Table 3:** Example quotes by theme for recall process used by mothers of 3–7-year-old children, and barriers and facilitators to recall (*n* = 21).

Theme	Example Quote
RECALL PROCESS USED BY PARENTS	
Information retrieval step one—filtering relevant information	**Food preferences**: *“He doesn’t eat a lot of meat. He doesn’t like it so looking at that list and the only thing is ham and fritz that he eats and I’ll offer ham probably a couple of times a week*…”—Mother 2 (SFS Discretionary) **Usual routine (recall of core foods ^a^)**: *“I think just generally thinking about the schedule of the week, so what day we had people over, what we do every night of the week. My husband has basketball on Tuesday night, so that’s what triggered for me the memory of what we had for dinner that night. ”*—Mother 9 (PDQ Grains) **Disrupted routine (recall of discretionary foods ^b^)**: *“…I was thinking what are the unusual circumstances that would have led to her eating those kind of things”*—Mother 13 (PDQ discretionary) **Meal and snack times:** *“…I’m thinking of dinners. That’s when we normally eat all of our vegies. And I’m also thinking of lunches. Main meals is our vegie intake.”*—Mother 2 (CDQ vegetables)
Information retrieval step two—use of specific memory prompts	**Occasions, events, treats and activities:** *“We wouldn’t just have them at home… So I’m trying to think how often we have an occasion that is linked to that type of treat.”*—Mother 4 (SFS discretionary) **Grandparents and other care givers:** *“I have a feeling that my husband took him out Saturday and gave him one [pie, pasty or sausage roll].”*—Mother 11 (CDQ discretionary) *“See this [cakes, biscuits, buns, muffins] is a grandparent’s specialty. So I suspect that he would probably have had them three times in the past week.”* – Mother 3 (PDQ discretionary) **Meal preparation:** *“We would probably have spaghetti bolognaise once a week, but he doesn’t eat the meat. We would have a roast probably once a week where he does eat the meat. And we would have chops or stir fry and he would eat the chops but not the stir fries.”*—Mother 17 (SFS meat) **Children’s response to food:** *“I’m thinking about how often I serve them against how much she actually consumes of them. I’d be wanting to say each day; she doesn’t eat them every day. But I give them to her every day.”*—Mother 5 (SFS vegetables) *“I was also thinking about what he actually ate. Because with the lettuce I know that he pulled some of the lettuce out and put it on my plate.”*—Mother 16 (CDQ vegetables) **Home food availability (core foods):** *“Knowing what sort of foods we have in the, in the fridge, in the pantry, again I’m very consistent with that.”*—Mother 13 (PDQ vegetables) **Home food availability and accessibility (discretionary foods):** *“Sweet biscuit dishes are high and still pre stocked so they haven’t snuck into there. Although she did take some biscuits, sweet biscuits today.”*—Mother 15 (PDQ discretionary) **Food purchasing:** *“I’m trying to remember what was in the shopping trolley because that’s probably what we actually ended up eating and what’s in the fridge.”*—Mother 18 (CDQ vegetables)
BARRIERS AND FACILITATORS OF REPORTING CHILDREN’S DIETARY INTAKE	
Understanding of foods to report	*“We do our own chips. So we get a potato, cut it and cook it on the barbie and we call them chips…Is that included as a homemade chip, it’s similar to a roast? It’s not deep fried, its maybe got a little bit of olive oil on it.”*—Mother 3 (PDQ discretionary)
Specificity of questions	*“It’s good having the little prompts saying including this, because that makes me think about what he would eat, and what he wouldn’t eat.”*—Mother 14 (PDQ discretionary)
Reporting portion size	**Estimating in units:** *“Like the chocolate, two pieces, two to three pieces, seven to fourteen pieces, that’s really clear, it’s easy to work out. But if it said a hundred grams of chocolate I’d be like, well I don’t know how much a hundred grams is.”*—Mother 13 (PDQ discretionary) **Cognitive load of converting intake to response option:** *“We took a whole packet in the boat but there was seven of us that shared it so I’m trying to think of how that would have divvied out. [Pause] It’s probably, I don’t know, a hundred- and eighty-gram bag, those big ones I think…so divide that by seven. It’s more than twenty grams each”*—Mother 15 (PDQ discretionary) **Guesstimating portion size**: *“So, again, I’d be guessing. I have no idea about how many grams of chips [laughs] she consumes… I’d be guessing but I think I’d go for the middle one, half to one, small packet. I’m guessing on that one because it’s in the middle so it’s the average.”*—Mother 5 (PDQ discretionary)
Recall time frame	*“I can actually think of the last seven days and answer it with some confidence.”*—Mother 19 (CDQ discretionary)
Recall difficulties	*“I can remember one specific time of cooking read meat, and I just don’t know whether I cooked it again. So, I couldn’t really tell you between once or two to four times at this point in time. I would err on the side of safety and say it’s two to four times.”*—Mother 1 (PDQ meat)
Unsure of foods child consumes when away from parents	*“The complication around this is that I’m looking at dinner meals but I know that my son is at an early learning centre and they also have cooked meals, not just sandwiches but I have no idea what regularly they have… So that’s the thing I’m just completely disregarding…”*—Mother 3 (SFS meat)
Frequency versus portion size	*“We’ve had quite a lot of salad this week, but he’s not too keen on lettuce…I’d probably tick yes, he’s had it, but it would be a miniscule amount.”*—Mother 16 (CDQ vegetables)
Child fussy eating	*“So that’s difficult because I can easily remember when I offer. It’s harder to remember what they actually eat because I don’t write it down or catalogue it.”*—Mother 18 (CDQ vegetables)
Reporting ‘usual’ intake	*“We’ve had [takeaway] a lot more in the last week than we usually would. So I’m wondering should I do what would be an average week or what has been the last week?”*—Mother 6 (SFS discretionary)
Social desirability	*“…we have Coco Pops in the school holidays, so if we’re talking the past seven days, that doesn’t look so good, does it?”*—Mother 9 (CDQ discretionary) *“It was confronting (laughs) because I had to admit to myself how many times I feed kids the foods I tell myself I’m not going to feed the kids.”*—Mother 4 (SFS discretionary) *“The only thing is my own value set, it’s getting a bit high, I don’t really like that idea. I’ll have to circle it anyway. That’s where I found the difficulty. Down here was easy because it’s nice, I’m feeling okay about that.”*—Mother 11 (CDQ discretionary)

**^a^** Core food groups: grains, meat and alternatives (‘meat’), vegetables. **^b^** Discretionary foods: ‘unhealthy’ foods, higher in saturated fat, added sugars and/or salt. Abbreviations: CDQ—Child Diet Questionnaire; PDQ—Pre-schooler Dietary Questionnaire; SFS—Short Food Survey.

**Table 4:** Coding of response strategies reported in cognitive interviews with mothers (*n* = 21) when reporting children’s dietary intake according to the taxonomy of Conrad et al. [8]

Response Strategy	Definition	Example Quote
Enumeration	Parent explicitly counted the number of times the child ate a particular food.	*“She had them the day before, so that’s 1, and then Friday she didn’t have them. Thursday I think she had them before school, so that’s 2, and I bought them on Tuesday night and she probably had them Wednesday morning, so that’s 3.”*—Mother 9 (CDQ discretionary)
Rate retrieval	Parent directly recalled the rate from memory, as opposed to estimating it.	*“I’d go 7 to cover breakfast for sure. We always have cereal.”*—Mother 2 (PDQ grain)
Rate estimation	Parent actively generated the rate, as opposed to directly retrieving it from memory.	*“So, I’d say probably on average, maybe two times a week.”*—Mother 9 (SFS meat)
Rate and adjustment	Parent indicated use of rate information (either though rate retrieval or rate estimation) but then adjusted the rate up or down to account for exceptions.	*“Well, if I think about the day she’s at home with me on a Friday then it kinda probably goes up a little bit to maybe to 16 rather than 14”*—Mother 1 (SFS grain)
General impressions	Parent first used language related to the magnitude of their child’s liking of a food, then converted that subjective impression to a specific rate.	*“He loves cucumber…he would have that more than five times”*—Mother 11 (PDQ vegetables)

Core food groups: grains, meat and alternatives (‘meat’), vegetables. Discretionary foods: ‘unhealthy’ foods, higher in saturated fat, added sugars and/or salt. Abbreviations: CDQ—Child Diet Questionnaire; PDQ—Pre-schooler Dietary Questionnaire; SFS—Short Food Survey.

## Appendix A

**Table A1:** Interview guide used in cognitive interviews with mothers (*n* = 21) of 3–7-year-old children.

**THINK-ALOUD PRACTICE EXAMPLE**
“Try to visualize the place where you live and think about how many windows there are in that place. As you count the windows, tell me what you are seeing and thinking about to work out the number of windows.”
**THINK-ALOUD**
“Now I would like you to read each question out loud, and as you are deciding on your answer say everything that you are thinking.” Prompts if needed: What are you thinking? What are you thinking about right now? Tell me what you’re thinking. Read the question out loud.
**SEMI-STRUCTURED INTERVIEW GUIDE**
GENERAL: How did the participant arrive at the answer? What did you think about to work out your answer? I noticed that you hesitated…What were you thinking about? Can you tell me in your own words what you think this question means?
COMPREHENSION: How was the question/food group interpreted? What foods did you include/consider when you were thinking about [X food group]? What do you understand by [X food group]? Was there anything that you didn’t understand? Were there any foods that you weren’t sure about whether to include or exclude from your answer?
RECALL: Recall processes used to arrive at the answer What did you think about when trying to recall that [your child] had [x foods]? What foods/meal occasions did you think about to determine/calculate your answer? Was there anything else you thought about when trying to work out how much/how often [child] eats [X]?
CONFIDENCE: How sure are you of your answer?
DIFFICULTY OF QUESTION: How easy or difficult did you find this question to answer?
RESPONSE OPTIONS: Were you able to find the right answer from the response options given?
**QUESTIONNAIRE SPECIFIC FOLLOW-UP QUESTIONS FOR PDQ—PORTION SIZE**
How did you think about/work out portion size? Were the portion size responses appropriate for your child? How easy or difficult did you find it to consider the serving sizes specified for [X food group]? How does the portion (amount) that your child usually eats compare to the serving sizes that are specified in the question?

**Abbreviations**: PDQ = Pre-schooler Dietary Questionnaire.

## References

[1] Abarca-Gómez L., Abdeen Z.A., Hamid Z.A., Abu-Rmeileh N.M., Acosta-Cazares B., Acuin C., Adams R.J., Aekplakorn W., Afsana K., Aguilar-Salinas C.A. (2017). Worldwide trends in body-mass index, underweight, overweight, and obesity from 1975 to 2016: A pooled analysis of 2416 population-based measurement studies in 128·9 million children, adolescents, and adults. Lancet.

[2] Waters E., de Silva-Sanigorski A., Burford B.J., Brown T., Campbell K.J., Gao Y., Armstrong R., Prosser L., Summerbell C.D. (2011). Interventions for preventing obesity in children. Cochrane Database Syst. Rev..

[3] Byrne R., Bell L., Taylor R.W., Mauch C., Mihrshahi S., Zarnowiecki D., Hesketh K.D., Wen L.M., Trost S.G., Golley R. (2019). Brief tools to measure obesity-related behaviours in children under 5 years of age: A systematic review. Obes. Rev..

[4] Magarey A., Watson J., Golley R.K., Burrows T., Sutherland R., McNaughton S.A., Denney-Wilson E., Campbell K., Collins C. (2011). Assessing dietary intake in children and adolescents: Considerations and recommendations for obesity research. Int. J. Pediatric Obes..

[5] McPherson R.S., Hoelscher D.M., Alexander M., Scanlon K.S., Serdula M.K. (2000). Dietary Assessment Methods among School-Aged Children: Validity and Reliability. Prev. Med..

[6] Golley R.K., Bell L.K., Hendrie G.A., Rangan A.M., Spence A., McNaughton S.A., Carpenter L., Allman-Farinelli M., Silva A., Gill T. (2017). Validity of short food questionnaire items to measure intake in children and adolescents: A systematic review. J. Hum. Nutr. Diet..

[7] Hendrie G.A., Viner Smith E., Golley R.K. (2014). The reliability and relative validity of a diet index score for 4–11-year-old children derived from a parent-reported short food survey. Public Health Nutr..

[8] Conrad F.G., Brown N.R., Cashman E.R. (1998). Strategies for Estimating Behavioural Frequency in Survey Interviews. Memory.

[9] Subar A.F., Freedman L.S., Tooze J.A., Kirkpatrick S.I., Boushey C., Neuhouser M.L., Thompson F.E., Potischman N., Guenther P.M., Tarasuk V. (2015). Addressing current criticism regarding the value of self-report dietary data. J. Nutr..

[10] Schwarz N. (2007). Cognitive aspects of survey methodology. Appl. Cogn. Psychol..

[11] Tourangeau R., Rips L.J., Rasinski K. (2000). The Psychology of Survey Response.

[12] Collins D. (2003). Pretesting survey instruments: An overview of cognitive methods. Qual. Life Res..

[13] Blair E., Burton S. (1987). Cognitive processes used by survey respondents to answer behavioral frequency questions. J. Consum. Res..

[14] Burton S., Blair E. (1991). Task conditions, response formulation processes, and response accuracy for behavioral frequency questions in surveys. Public Opin. Q..

[15] Drennan J. (2003). Cognitive interviewing: Verbal data in the design and pretesting of questionnaires. J. Adv. Nurs..

[16] Carbone E.T., Campbell M.K., Honess-Morreale L. (2002). Use of cognitive interview techniques in the development of nutrition surveys and interactive nutrition messages for low-income populations. J. Am. Diet. Assoc..

[17] Johnson-Kozlow M., Matt G.E., Rock C.L. (2006). Recall strategies used by respondents to complete a food frequency questionnaire: An exploratory study. J. Am. Diet. Assoc..

[18] Wolfe W.S., Frongillo E.A., Cassano P.A. (2001). Evaluating brief measures of fruit and vegetable consumption frequency and variety: Cognition, interpretation, and other measurement issues. J. Am. Diet. Assoc..

[19] Banna J.C., Becerra L.E.V., Kaiser L.L., Townsend M.S. (2010). Using qualitative methods to improve questionnaires for Spanish speakers: Assessing face validity of a food behavior checklist. J. Am. Diet. Assoc..

[20] Willis G.B., Royston P., Bercini D. (1991). The use of verbal report methods in the development and testing of survey questionnaires. Appl. Cogn. Psychol..

[21] Willis G.B. (2005). Cognitive Interviewing. A Tool for Improving Questionnaire Design.

[22] Australian Bureau of Statistics (2014). 4364.0.55.007—Australian Health Survery: Nutrition First Results—Food and Nutrients, 2011–2012.

[23] Saunders B., Sim J., Kingstone T., Baker S., Waterfield J., Bartlam B., Burroughs H., Jinks C. (2018). Saturation in qualitative research: Exploring its conceptualization and operationalization. Qual. Quant..

[24] National Health and Medical Research Council (2013). Australian Dietary Guidelines.

[25] Department of Health Discretionary Food and Drink Choices. https://www.eatforhealth.gov.au/food-essentials/discretionary-food-and-drink-choices.

[26] Magarey A., Golley R., Spurrier N., Goodwin E., Ong F. (2009). Reliability and validity of the Children’s Dietary Questionnaire; a new tool to measure children’s dietary patterns. Int. J. Pediatric Obes..

[27] Bell L.K., Golley R.K., Mauch C.E., Mathew S.M., Magarey A.M. (2018). Validation testing of a short food-group-based questionnaire to assess dietary risk in preschoolers aged 3–5 years. Nutr. Diet..

[28] Janghorban R., Roudsari R.L., Taghipour A. (2014). Skype interviewing: The new generation of online synchronous interview in qualitative research. Int. J. Qual Stud. Health Well-Being.

[29] (2015). QSR International Pty Ltd. NVIVO, 11.

[30] Liamputtong P. (2009). Qualitative data analysis: Conceptual and practical considerations. Health Promot. J. Aust..

[31] Braun V., Clarke V. (2006). Using thematic analysis in psychology. Qual. Res. Psychol..

[32] Harris J.E., Gleason P.M., Sheean P.M., Boushey C., Beto J.A., Bruemmer B. (2009). An introduction to qualitative research for food and nutrition professionals. J. Am. Diet. Assoc..

[33] Subar A.F., Thompson F.E., Smith A.F., Jobe J.B., Ziegler R.G., Potischman N., Schatzkin A., Hartman A., Swanson C., Kruse L. (1995). Improving food frequency questionnaires: A qualitative approach using cognitive interviewing. J. Am. Diet. Assoc..

[34] Matt G.E., Rock C.L., Johnson-Kozlow M. (2006). Using recall cues to improve measurement of dietary intakes with a food frequency questionnaire in an ethnically diverse population: An exploratory study. J. Am. Diet. Assoc..

[35] Dickin K.L., Lent M., Lu A.H., Sequeira J., Dollahite J.S. (2012). Developing a Measure of Behavior Change in a Program to Help Low-Income Parents Prevent Unhealthful Weight Gain in Children. J. Nutr. Educ. Behav..

[36] Townsend M.S., Shilts M., Ontai L., Leavens L., Davidson C., Sitnick S. (2014). Obesity risk for young children: Development and initial validation of an assessment tool for participants of federal nutrition programs. Forum Fam. Consum. Issues (FFCI).

[37] Lindsay A.C., Sussner K.M., Greaney M., Wang M.L., Davis R., Peterson K.E. (2012). Using Qualitative Methods to Design a Culturally Appropriate Child Feeding Questionnaire for Low-Income, Latina Mothers. Matern. Child. Health J..

[38] Thoyre S.M., Pados B.F., Park J., Estrem H., Hodges E.A., McComish C., Van Riper M., Murdoch K. (2014). Development and content validation of the pediatric eating assessment tool (Pedi-EAT). Am. J. Speech Lang. Pathol..

[39] Menon G., Yorkston E.A., Stone A.A., Bachrach C.A., Jobe J.B., Kurtzman H.S., Cain V.S. (1999). The use of memory and contextual cues in the formation of behavioral frequency judgements. The Science of Self-Report: Implications for Research and Practice.

[40] Tversky A., Kahneman D. (1973). Availability: A heuristic for judging frequency and probability. Cogn. Psychol..

[41] Domel S.B., Thompson W.O., Baranowski T., Smith A.F. (1994). How children remember what they have eaten. J. Am. Diet. Assoc..

[42] Rhodes K., Chan F., Prichard I., Coveney J., Ward P., Wilson C. (2016). Intergenerational transmission of dietary behaviours: A qualitative study of Anglo-Australian, Chinese-Australian and Italian-Australian three-generation families. Appetite.

[43] Menon G. (1997). Are the parts better than the whole? The effects of decompositional questions on judgments of frequent behaviors. J. Mark. Res..

